# Phytochemical Profiles and Biological Activities of Plant Extracts from Aromatic Plants Cultivated in Cyprus

**DOI:** 10.3390/biology13010045

**Published:** 2024-01-15

**Authors:** Antonios Chrysargyris, Jovana D. Petrovic, Ekaterina-Michaela Tomou, Kalia Kyriakou, Panayiota Xylia, Andria Kotsoni, Vasiliki Gkretsi, Panagiota Miltiadous, Helen Skaltsa, Marina D. Soković, Nikolaos Tzortzakis

**Affiliations:** 1 Department of Agricultural Sciences, Biotechnology and Food Science, Cyprus University of Technology, Limassol 3036, Cyprus; 2 Institute for Biological Research “Siniša Stanković” - National Institute of Republic of Serbia, University of Belgrade, Bulevar despota Stefana 142, 11108 Belgrade, Serbia; jovana0303@ibiss.bg.ac.rs (J.D.P.);; 3 Department of Pharmacognosy & Chemistry of Natural Products, School of Health Sciences, Faculty of Pharmacy, National and Kapodistrian University of Athens, Panepistimiopolis, Zografou, 15771 Athens, Greece; 4 Department of Nursing, School of Health Sciences, Cyprus University of Technology, Limassol 3041, Cyprus; kalia.kyriakou@cut.ac.cy (K.K.);; 5 Department of Life Sciences, School of Sciences, European University Cyprus, Nicosia 2404, Cyprus; ak222430@students.euc.ac.cy (A.K.); v.gkretsi@euc.ac.cy (V.G.); 6 Cancer Metastasis and Adhesion Group, Basic and Translational Cancer Research Center (BTCRC), European University Cyprus, Nicosia 2404, Cyprus

**Keywords:** chemical profiles, NMR, antioxidant activity, antimicrobial activity, cytotoxicity

## Abstract

**Simple Summary:**

Plant extracts and essential oils have been used for medicinal purposes since ancient times. Medicinal and aromatic plants (MAPs) are composed of various phytochemical and nutritive compounds with strong biological properties, including antioxidant, antimicrobial, and cytotoxic effects. These activities are often related to the synergistic effects of compounds other than the predominant molecule. Exploitation of endemic species such as *Sideritis cypria* may give a boost to the MAP sector, with high pharmacological interest in green medicine production using natural components.

**Abstract:**

Medicinal and aromatic plants’ properties, still an interesting research area, are attributed to the presence of various specialized products that possess important pharmacological activities. In the present study, six medicinal/aromatic plants (*Sideritis cypria*, *Origanum dubium*, *Melissa officinalis*, *Mentha piperita*, *Thymus capitatus*, and *Salvia fruticosa*) were evaluated for their phytochemical and nutritive composition, as well as their biological activities, including antioxidant, antimicrobial, and cytotoxic properties. The results obtained indicate that *M. piperita* was rich in proteins and minerals such as N and Mg, while *S. cypria* accumulated more K, Na, P, and Ca. The highest content of phenols and flavonoids was observed in *M. piperita*, followed by *O. dubium* and *T. capitatus*, which eventually influenced their high antioxidant capacity. NMR screening revealed the presence of (i) triterpenoids and hydroxycinnamic acid derivatives in *M. officinalis*; (ii) terpenoids, flavonoids, and phenolic acid derivatives in *S. fruticosa*; (iii) flavonoids and phenolic acid derivatives in *M. piperita*; (iv) phenolic monoterpenes in *O. dubium* and *T. capitatus*; and (v) terpenoids, flavones, and phenylethanoid glycosides in *S. cypria*. The results of the antimicrobial activity showed that the tested samples overall had quite good antimicrobial potential. High antibacterial activity was found in *O. dubium* and *T. capitatus*, while *O. dubium* and *S. cypria* exhibited great antifungal activities. The studied species also had an important effect on the viability of female-derived and colon cancer cells. In particular, in colon cancer cells, the extracts from *T. capitatus, M. officinalis, M. piperita*, and *S. fruticosa* exhibited a stronger effect on cell viability in the more metastatic cell line at significantly lower concentrations, indicating an important therapeutic potential in targeting highly metastatic tumors. This finding is worth further investigation. The present study unveiled interesting phytochemical profiles and biological properties of the six medicinal/aromatic plants, which should be further explored, contributing to green chemistry and the possible creation of natural health products for humans’ health/nutrition and additives in cosmetics.

## 1. Introduction

Medicinal and aromatic plants (MAPs) have been used, long before the advent of pharmaceutical products, to prevent and treat a broad range of diseases, as well as to improve people’s well-being [1]. These plants, still an interesting research area, are a rich source of substances and bioactive compounds with many pharmacological activities and a limited number of side effects [2]. More specifically, their beneficial properties could be attributed to their valuable content of nutrients and phytochemical compounds like polyphenols, which contribute to the management, treatment, and prevention of a wide range of pathological conditions, including cancer, inflammation, liver injuries, and oxidative stress [3]. Furthermore, contemporary dietary patterns also enlist MAPs as a source of functional food, which offers additional benefits beyond the common nutritional requirements [4].

MAPs may be consumed as fresh or dried material [5,6], as frozen [7], or they may be added as antimicrobial agents and natural food preservatives to inhibit the growth of pathogenic microorganisms related to food spoilage [8] and/or as anti-oxidants to reduce the oxidative deterioration of food [9]. This has an imminent effect on multiple applications in the food supply chain, ranging from producers and distributors to consumers [9,10]. From this viewpoint, plants used daily as beverages or spices are of particular interest. Over the years, global use and trade of MAPs or MAP-derived products have been dramatically expanded, highlighting their importance as well as the necessity of supporting the conservation of each region’s native populations, with that being especially necessary for the entire region of the Mediterranean Sea [1].

The area of the Mediterranean basin is characterized by native plant species with numerous ecotypes. More than 10,000 different species of MAPs have been identified to date [11], while more than 50% of the vascular plants of the Mediterranean are exclusive to the region [12]. The phytochemical composition of MAPs could vary greatly and significantly depending on several factors, including the climate conditions (geo-climatic and seasonal changes, temperature, light, humidity, and developmental processes), the cultivation practices followed, and the genetic composition of each plant [4,13]. As a result, the pharmacological properties could also be affected. The Mediterranean region has been recently characterized as a complex area with multivariable geographical and ecological patterns that has an ongoing history of plant population evolution as it combines microhabitats and geographical isolation (i.e., islands) and includes many hot spots with high endemic and/or indigenous species and cultivars [12,14].

Within this context, Cyprus, as the 3rd largest island in the Mediterranean basin, perfectly fits this profile. Additionally, the island’s climate conditions (high temperatures, winds, and drought) provide the cultivation of local MAP species with promising prospects as crops that have low requirements for irrigation water and energy while increasing their nutritional and biological value [4]. Thus, the present study aimed to produce cultivated added value MAP species (indigenous, endemic, or not) that are most commonly used and traded in Cyprus, focusing on their phytochemical composition and the antimicrobial, antioxidant, and cytotoxic properties of their extracts, contributing to increasing the therapeutic usefulness of natural pharmaceuticals along with fulfilling pharmacopoeial standards and consumer/industry needs.

## 2. Materials and Methods

### 2.1. Plant Material

Plant material was collected from the plantations of the Department of Agriculture (Medicinal and Aromatic Plants Section) of the Cyprus Ministry of Agriculture, Rural Development, and Environment (33.135685, 33.403125). Aerial parts of six different species (*Sideritis cypria* Post., *Origanum dubium* Boiss., *Melissa officinalis* L., *Mentha piperita* L., *Thymus capitatus* (L.) Hoffmanns & Link, and *Salvia fruticosa* Mill.) were collected on 18th and 19th of July 2022. The plants were identified by staff members of the Cypriot National Agricultural Department. Approximately 4.0 kg of fresh aerial parts of the species were harvested at the flowering stage and transferred to the laboratories of the Cyprus University of Technology. After sampling the fresh tissue for the antioxidant activity analysis, plant tissue was dried in an air oven at 42 °C until constant weight. The dried material was then milled into a fine powder.

### 2.2. Mineral Analysis and Nutritional Value

The mineral content of the aerial parts of the plants was analyzed using four replicates. The dried material was ashed in a furnace (Carbolite, AAF 1100, GERO, Neuhausen, Germany) at 450 °C for 6 h and then digested using 2 N HCl [15]. The produced extract was used for the determination of phosphorus (P) content (molybdate/vanadate method), while potassium (K), sodium (Na), calcium (Ca), and magnesium (Mg) were measured using ion chromatography (ICS-3000, Dionex Aquion, Sunnyvale, CA, USA) equipped with an IonPac CS19 (4 × 250 mm, Dionex, Corporation, Sunnyvale, CA, USA) analytical column [15]. Nitrogen (N) content was determined by the Kjeldahl method (BUCHI, Digest automat K-439, and Distillation Kjeldahl K-360, Essen, Deutschland) [15]. Data were expressed in g/kg of dry weight.

The nutritional value of each species was determined using the AOAC techniques after the assessment of moisture, protein, fat, carbohydrates, and ash composition [16]. Briefly, nutrient content was determined by using the Kjeldahl (N × 6.25), petroleum ether Soxhlet extraction, and incineration (600 °C) methods for protein, fat, and moisture, respectively. The carbohydrate content was determined by the difference in dry weight, and the energetic value was estimated by applying the following Formula (1):

Energy (kcal/100 g dried tissue) = 4 × (g of protein/100 g + g of carbohydrate/100 g) + 9 × (g of fat/100 g) (1)

Results were expressed as g/100 g of dried tissue.

### 2.3. Phytochemical Analysis

#### 2.3.1. Extraction and NMR Analysis

For the phytochemical profile analysis, 40 g of dried aerial parts of each plant were separately extracted at room temperature with methanol (3 × 24 h). Then, they were filtered and concentrated to dryness to yield residues of 4.6 g (*M. officinalis*), 9.6 g (*S. fruticosa*), 6.0 g (*M. piperita*), 5.3 g (*O. dubium*), 3.6 g (*T. capitatus*), and 5.0 g (*S. cypria*).

For NMR experiments, a part of each sample (10.0 mg) was dissolved in 600 μL of CD_3_OD. One- and two-dimensional NMR spectra were recorded on a Bruker 400 DRX instrument at 300 K. Chemical shifts are given in ppm (δ) and are referenced to the solvent signal at 3.31/49.0 ppm (CD_3_OD) for ^1^H- and ^13^C-NMR, respectively. COSY (COrrelation SpectroscopΥ) and HSQC (heteronuclear single quantum coherence) experiments were performed using standard Bruker microprograms.

#### 2.3.2. Preparation of Plant Extracts

Ethanolic extracts from the tested species were prepared as previously described [16]. The plant material was then macerated with pure ethanol (1:2.5 *v*/*v*) in a shaking incubator at 160 rpm, in glass bottles. The material was filtered using filter paper after 72 h of shaking. Using a rotary evaporator unit, the ethanolic extract was then concentrated to dryness and yielded. The produced extracts from the six tested species were then used for the evaluation of their biological activities.

#### 2.3.3. Total Phenolic and Flavonoid Content and Antioxidant Activity

The total phenolic content was determined by using the Folin–Ciocalteu method, according as described previously [15,17]. Plant extracts were mixed with 125 µL of Folin–Ciocalteu reagent and 1.25 mL of 7% Na_2_CO_3_. After incubation in the dark for 60 min, the absorbance of the reactions was measured at 755 nm. Total phenolic content was expressed as µmol of gallic acid equivalents per gram of dried extract (µmol GAE/g DE). Total flavonoid content was assayed using the aluminum chloride colorimetric method [18]. Plant extracts were mixed with 0.75 mL of 5% sodium nitrite (NaNO_2_) and incubated for 6 min. Then, 0.15 mL of AlCl_3_ solution (10%) was added, and after an additional 5 min, 0.5 mL of NaOH (1 M) solution was added. The absorbance of the reaction mixture was measured at 510 nm, using rutin as a standard. Results were expressed as rutin equivalents (mg rutin/g DE).

The total antioxidant activity was assessed by the 2,2-diphenyl-1-picrylhydrazyl (DPPH), the ferric reducing antioxidant power (FRAP), and the 2,2′-azino-bis(3-ethylbenzothiazoline-6-sulphonic acid (ABTS) methods, as described previously [4,19]. Briefly, the DPPH radical scavenging activity of the extracts was measured at 517 nm, while the FRAP activity was measured at 593 nm. The ABTS assay was implemented according to the methodology described by Woidjylo et al. [20]. Results were expressed as Trolox ((±)-6-Hydroxy-2,5,7,8-tetramethylchromane-2-carboxylic acid) equivalents (mg trolox/g of DE).

### 2.4. Antimicrobial Activity

#### 2.4.1. Microorganisms and Cultivation Media

The following Gram (+) bacteria: *Staphylococcus aureus* (ATCC 11632), *Bacillus cereus* (food isolate), and *Listeria monocytogenes* (NCTC 7973), as well as Gram (-) bacteria *Escherichia coli* (ATCC 25922) and *Salmonella enterica* subsp. *enterica* serovar Typhimurium (ATCC 13311) were tested; as for the tested micromycetes, the following were used: *Aspergillus fumigatus* (ATCC 9197), *Aspergillus niger* (ATCC 6275), *Aspergillus versicolor* (ATCC 11730), *Penicillium funiculosum* (ATCC 36839), *Penicillium verrucosum* var. *cyclopium* (food isolate), and *Trichoderma viride* (IAM 5061). The microorganisms are deposited at the Mycological Laboratory, Department of Plant Physiology, Institute for Biological research, “Sinisa Stanković”, National Institute of the Republic of Serbia, University of Belgrade, Serbia.

#### 2.4.2. Microdilution Method

The antibacterial assay was carried out using a modified microdilution method [21]. Bacterial strains were cultured overnight at 37 °C in tryptic soy broth, after which they were adjusted with sterile saline to a concentration of 1.0 × 10^5^ CFU/mL. Samples dissolved in a 30% solution of ethanol in water were added to tryptic soy broth (TSB) medium (100 µL) with bacterial inoculum (1.0 × 10^4^ CFU per well). After incubation (24 h at 37 °C), *p*-iodonitrotetrazolium chloride (40 μL, 0.2 mg/mL) was added to each well of the plate and further incubated for 60 min at 37 °C for the color development. The lowest concentrations that showed a distinct reduction in color intensity—light red in comparison to the intensive red in the control well (with no added extract) or an absence of color—were defined as minimal inhibitory concentrations (MICs). The minimal bactericidal concentrations (MBCs) were determined by serial sub-cultivation of 2 µL into the wells already containing 100 µL of broth and further incubation for 24 h at 37 °C. The lowest concentration with no visible growth was defined as the MBC, indicating 99.5% killing of the original inoculum. Commercial preservatives E211 and E224 were used as positive controls. The experiments were performed in triplicate.

The antifungal assay was performed according to Stojkovic et al. [22]. Briefly, MIC and minimal fungicidal concentrations (MFCs) values were determined by a serial dilution technique using 96-well microtiter plates. Tested extracts were previously dissolved in a 30% solution of ethanol in water, while commercial preservatives E211 and E224 were used as positive controls, and experiments were performed in triplicate.

### 2.5. Cell Viability following Treatment with the Plant Extracts

#### 2.5.1. Cell Culture

A total of five human cancer cell lines were used: MDA-MB231 (highly invasive breast cancer cells), HeLa (cervical cancer cells), SKOV-3 (ovarian cancer cells), HT-29 (less invasive colorectal cancer cells), and HCT-116 (highly invasive colorectal cancer cells). MDA-MB231 and HeLa cell lines were maintained in culture flasks containing low-glucose Dulbecco’s modified Eagle’s medium (DMEM). SKOV3, HT-29, and HCT-116 cell lines were cultured in McCoy’s 5A medium. All media were supplemented with 10% Fetal Bovine Serum (FBS), 1% Penicillin/Streptomycin, and 0.05% Trypsin solution for subculturing. Reagents for cell culture were purchased from Invitrogen Life Technologies. All cells were incubated at 37 °C in a humidified atmosphere of 5% CO_2_.

#### 2.5.2. MTT Assay

Ethanolic extracts were weighted and dissolved in 0.1% Dimethyl Sulfoxide (DMSO) (diluted with PBS) to obtain stock solutions with concentrations ranging from 200–1000 μg/mL. Cell viability following treatment with the plant extracts was assessed using the MTT (3-(4,5-dimethylthiazol-2-yl)-2,5-diphenyltetrazolium bromide) cell viability assay [23]. Briefly, MDA-MB231, HeLa, SKOV3, HT-29, and HCT-116 cells were seeded in triplicate in 96-well plates at a density of 10^4^ cells/well. Twenty-four (24) hours later, cells were treated with various concentrations of the ethanolic plant extracts (final concentrations of 200, 450, and 1000 μg/mL) for 48 or 72 h. Following completion of the 48 or 72 h, 10 μL of MTT were added to each well, while an equal amount of 0.1% DMSO was used to treat cells that served as the negative control, and the plate was incubated for 4 h at 37 °C. The MTT solution was then removed, and formazan crystals were dissolved by adding 200 μL of DMSO. Absorbance was then measured at a wavelength of 570 nm using a microplate reader (specifically, the ELx800TM Absorbance Microplate Reader (BioTek Instruments Inc., Santa Clara, CA, USA) for the experiments performed at the Cyprus University of Technology or the Thermo Scientific™ Varioskan™ LUX multimode microplate reader and SkanIt™ (Waltham, MA USA) for the experiments performed at the European University Cyprus). Half maximal inhibitory concentration (IC_50_) was calculated with the GraphPad Prism software 8.0 using dose response inhibition and a nonlinear regression curve fitting function. The individual concentration vs. normalized response data were used for the regression from at least three independent experiments. All MTT experiments were performed in triplicate, and at least three independent experiments were performed for each extract and time point. The data from the treatment condition of 1000 μg/mL at the time point of 72 h were used to generate cell viability.

### 2.6. Statistical Analysis

Measurements were performed in four to six biological replications/treatment (each replication consisted of a poll of two individual measures/samples). Statistical analysis was performed using the SPSS statistical software (SPSS v.22; IBM, Armonk, New York, NY, USA). Data means were also compared with one-way analysis of variance (ANOVA), and Duncan’s multiple range test and Student *t*-test were used for the comparison of treatment means at *p* < 0.05.

## 3. Results and Discussion

### 3.1. Mineral Content and Nutritional Value

Apart from being a major source of bioactive compounds, medicinal plants are vital ingredients in the human diet because of their high nutritional value. Minerals are crucial components of hormones, vitamins, enzyme activation systems, and/or cofactors in cellular metabolism [24]. A comparative mineral profile of the tested plants is illustrated in Table 1. The highest content of nitrogen (N) was found in *M. piperita* plants (20.55 g/kg), while the lowest was in *O. dubium* (11.46 g/kg); the N content for the rest of the species varies between 13.67 and 16.46 g/kg. The richer plant in potassium (K) (22.07 g/kg), calcium (Ca) (17.49 g/kg), and phosphorus (P) content (3.75 g/kg) was *S. cypria*, while the same species revealed the lowest content in magnesium (Mg) (2.30 g/kg). *M. piperita* also revealed significantly high levels of K, Mg, Ca, and P compared to the rest of the tested species. Interestingly, *O. dubium* and *M. officinalis* appeared to have the lowest content of sodium (Na), both at 0.13 g/kg, which is quite important as Na uptake is reported to be a determinant of hypertension, with reduced consumption being widely recommended [25]. In general, the results obtained revealed that the tested species are a great source of K, while at the same time their Na content is low. This seems like an important feature in water and electrolyte balance for various metabolic functions [26].

Plants play an important role in satisfying human needs for energy and nutrients. Carbohydrates, proteins, and fats, as well as minerals, are nutrients identified in plants that play an important role in establishing a balanced organ control system in humans [27]. The determination of the nutritional value of medicinal plants was investigated because fresh or dried MAPs have multiple purposes as infusions, decoctions, or extracts widely consumed worldwide. The values obtained for protein content in all the tested species are considered average according to the literature (lower than 20%) [24]. *M. piperita* had the highest protein content (12.84%), followed by *S. fruticosa* (10.28%); the latter was revealed to have the highest content of total fats (5.89%) (Table 2). The lowest fat values were observed in *T. capitatus* (0.69%), but the same species proved to have the highest content of carbohydrates (84.12%). Interestingly, the ash content appeared quite high in *S. cypria* (11.84%) and *M. piperita* (11.28%), with the rest being in the range of 6.30–8.18%. This is in accordance with the higher mineral content identified in these two species, as high ash content is an indication of a high level of inorganic minerals in a plant [24] and implies that these plants could act as a preservative (antimicrobial agent, as it will be discussed next) and digestion aid agent [28]. In general, even though significant differences have been identified in terms of caloric energy among the tested species, they all ranged from 367 to 401 kcal/100 g of dried material.

The differences in our results in comparison to previously published data in the literature could be associated with several attributes, such as the season and maturity stage of the plants, climate conditions, soil composition, and fertigation plan, to name a few [29]. The results obtained indicated that medicinal plants are overall a significant source of mineral composition. The nutrient analysis was within the range of other reports for *M. officinalis* [30], *S. cypria* [31], and *S. fruticosa* [32], or had small variations, as in the cases of *M. piperita* [33] or *T. capitatus* [34]. To our knowledge, this is the first report that analyzes the mineral content and nutritional value of *O. dubium*. Compared to other species of the *Origanum* genus, *O. dubium* appears to have a higher nutritional value than *O. compactum* [35].

### 3.2. NMR Analysis

**Figure 1:**
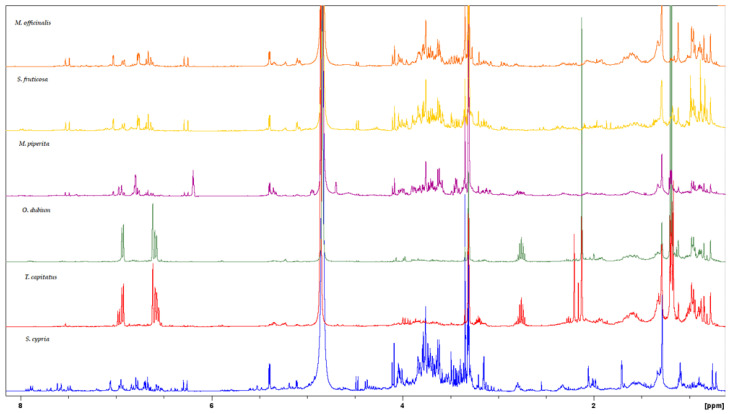
Overlaid ^1^H-NMR spectra of the methanol extracts of *M. officinalis* (orange color), *S. fruticosa* (yellow color), *M. piperita* (purple color), *O. dubium* (green color), *T. capitatus* (red color), and *S. cypria* (blue color).

A preliminary screening of the methanol extracts was first obtained by ^1^H-NMR spectra, and the phytochemical categories of their constituents were identified based on peaks in specific regions. The comparative 1D NMR fingerprints are presented in Figure 1. In addition, 2D NMR spectra (COSY and HSQC) were acquired in order to provide a better overview (Figures S1–S6).

**Figure 2:**
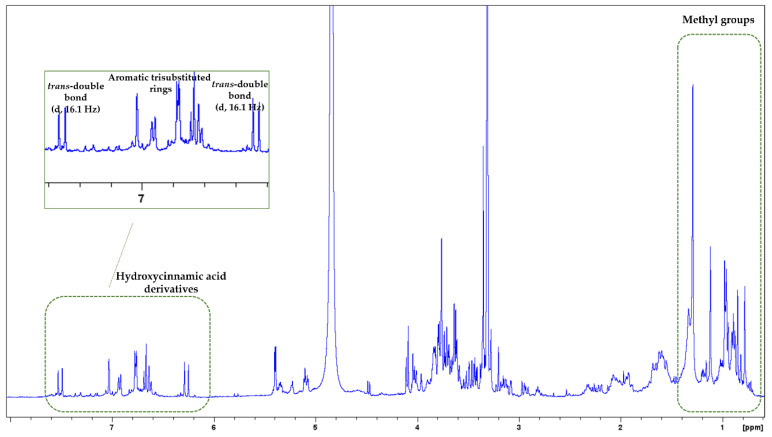
^1^H-NMR spectrum of *M. officinalis*. Signals from methyl groups and hydroxycinnamic acid derivatives are indicated with green boxes. Zoom-in of the aromatic region.

In the ^1^H-NMR spectrum of *M. officinalis*, mainly signals from triterpenoids and phenolic acid derivatives were observed (Figure 2). In the upfield region (2.80–0.70 ppm), proton signals appeared that might belong to triterpenoids. Precisely, signals of methyl groups of triterpenoids were found in the δ_H_ range of 1.30–0.77, while the corresponding carbon signals were spotted in the range of δc 33.0–14.0 in the HSQC spectrum (Figure S1a). In addition, overlapped multiple peaks suggest the presence of methine and methylene groups, which could also indicate the presence of triterpenoids. The second region (5.90–3.10 ppm) included signals, especially of sugars. It should be mentioned that some peaks in this region could also be assigned to the protons of triterpenoids and phenolic acid derivatives. Ιn the aromatic region (7.70–6.10 ppm), mainly peaks of phenolic acid derivatives were illustrated. Specifically, proton signals in this region could be assigned to hydroxycinnamic acid derivatives. At δ_H_ 7.51 (d, J = 16.1 Hz) and 6.27 (d, J = 16.1 Hz), *trans*-coupling olefinic signals ascribable to the double-bond of hydroxycinnamic acid derivatives were detected. In the HSQC spectrum, their corresponding carbons were spotted at δc 146.8 and 115.4, respectively. In addition, at δ_H_ range 7.05–6.60 (HSQC: δc 120.5–112.1) were observed signals that could belong to protons of aromatic trisubstituted rings. In the ^1^H-^1^H-COSY spectrum, spin systems among the specific protons could be detected (Figure S1b). Previous studies have reported the presence of triterpenoids (such as ursolic and oleanolic acids), flavonoids, and phenolic acids, especially hydroxycinnamic acid derivatives, in *M. officinalis* extracts (e.g., caffeic acid, chlorogenic acid, and rosmarinic acid) [36,37,38].

**Figure 3:**
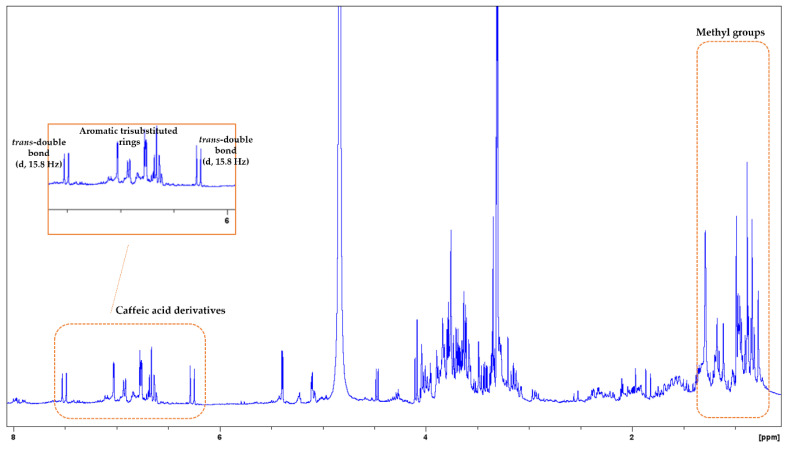
^1^H-NMR spectrum of *S*. *fruticosa.* Signals from methyl groups and caffeic acid derivatives are signed with orange boxes. Zoom-in of the aromatic region.

In the ^1^H-NMR spectrum of *S. fruticosa*, signals from terpenoids, flavonoids, and phenolic acid derivatives were detected (Figure 3). In the upfield region (2.80–0.70 ppm), proton signals of methyl, methine, and methylene groups belonging to terpenoids such as triterpenoids and diterpenoids appeared. The second region (5.90–3.10 ppm) included signals, especially of sugars. It should be mentioned that some peaks in this region could also be assigned to protons of terpenoids and phenolic acid derivatives. In the downfield region (8.00–6.00 ppm), there are mainly peaks of phenolic acid derivatives (especially caffeic acid monomers or/and oligomers), as well as flavonoids. Proton signals in this region could be assigned to caffeic acid monomers (e.g., caffeic acid, chlorogenic acid, and 3-(3,4-dihydroxyphenyl)lactic acid), caffeic acid oligomers (e.g., rosmarinic acid and salvianolic acids), and flavonoids (e.g., luteolin glycosides or its glucuronides). At δ_H_ 7.51 (d, J = 15.8 Hz) and 6.27 (d, J = 15.8 Hz), *trans*-coupling olefinic signals were observed, ascribable to the double bond of caffeic acid derivatives. In the HSQC spectrum (Figure S2a), their corresponding carbons were spotted at δc 145.3 and 114.2, respectively. In addition, at δ_H_ range 7.05–6.60 (HSQC: δc 121.6–113.7) were observed signals that could belong to protons of aromatic trisubstituted rings. In the ^1^H-^1^H-COSY spectrum, spin systems among the specific protons could be detected (Figure S2b). Triterpenoids and diterpenoids were previously detected in *Salvia* species [39,40]. In general, phenolic acid derivatives commonly occur in the genus *Salvia* [39,41,42]. Rosmarinic acid is the most predominant caffeic acid dimer, while other caffeic acid oligomers like salvianolic acids A-K and lithospermic acid are widely found in these species. Moreover, luteolin glycosides and their glucuronides are reported to be more common compared to those of apigenin in *Salvia* taxa [41].

**Figure 4:**
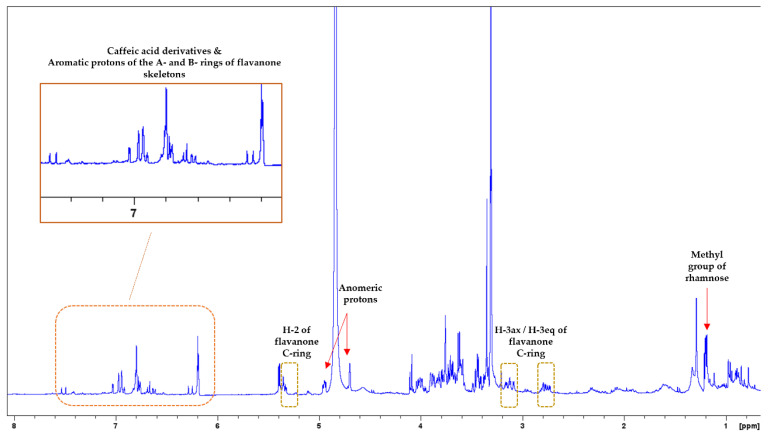
^1^H-NMR spectrum of *M. piperita.* Characteristic flavanone proton signals for H-3ax/H-3eq and H-2 of the C-ring are signed with brown boxes. Zoom-in of the aromatic region.

In the ^1^H-NMR spectrum of *M. piperita*, mainly signals from flavonoids, and phenolic acid derivatives were observed (Figure 4). In the aromatic region (7.60–6.10 ppm), proton signals could be assigned to (i) caffeic acid derivatives [δ_H_: 7.51 (d, J = 16.2) and 6.27 (d, J = 16.2)/δ_H_ range: 7.05–6.61], and (ii) flavonoids (δ_H_ range: 6.96–6.78 and 6.20–6.17). Regarding the flavonoids, the observation of double doublet peaks at δ_H_ range of 3.19–2.70, and δ_H_ 5.35 leads to the assumption of the presence of flavanones. These assignments are characteristic flavanone proton signals for H-3ax/H-3eq, and H-2 of the C-ring, respectively. In the ^1^H-^1^H-COSY spectrum, the correlation peaks between the above protons were also observed (Figure S3a). Their corresponding carbon signals were recorded at δc 43.9 (C-3) and 80.4 (C-2) based on the HSQC spectrum (Figure S3b). At the δ_H_ range of 6.20–6.17 and 6.96–6.78, signals could be assigned to aromatic protons of the A- and B-rings of flavanone skeletons. Furthermore, signals of sugars were observed in the middle region (5.40–3.30 ppm) of the ^1^H-NMR spectrum. Indeed, anomeric protons were found at δ_H_ ca. 4.95–4.93 (d, J = 7.9 Hz; HSQC: δc ca.100.9–100.8) and 4.69 (br s; HSQC: δc 101.9), as well as at δ_H_ around 1.20, a double peak (J = 6.1 Hz; HSQC: δc 17.7) could be attributed to a methyl group of rhamnose. Thus, we could assume that flavanones could be presented in their glycoside forms. Previous studies reported that *M. piperita* is rich in flavanones, especially eriodictyol, naringenin, and hesperidin derivatives [43,44]. It is noteworthy to mention that eriocitrin (eriodictyol-7-rutinoside) is one of the major compounds of flavanone derivatives of *M. piperita* [43,44]. On the contrary, flavonols and dihydroflavonols have been reported less in previous studies [43]. Phenolic acids, mainly caffeic acid and its derivatives (e.g., chlorogenic acid and rosmarinic acid), are found in *M. piperita* extracts [43,44].

**Figure 5:**
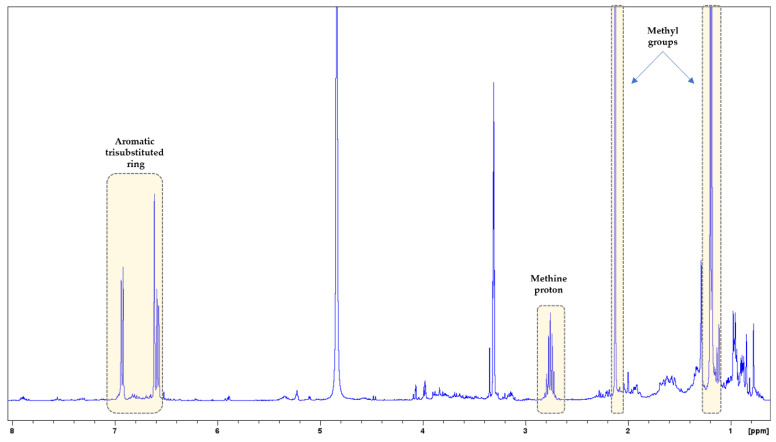
^1^H-NMR spectrum of *O. dubium.* Signals of phenolic monoterpene are indicated with yellow boxes.

In the NMR spectra of *O. dubium*, signals from terpenoids, flavonoids, and phenolic acids were observed. However, signals of phenolic monoterpene were mainly detected in the ^1^H-NMR spectrum: (i) aromatic protons of trisubstituted ring at δ_H_ 6.93 (d, J = 7.8 Hz), 6.62 (d, J = 1.5 Hz), and 6.58 (dd, J = 7.8/1.5 Hz); (ii) methine proton at 2.76 (sep); and (iii) methyl protons at 2.13 (s) and 1.19 (d, J = 6.9 Hz) (Figure 5). In the ^1^H-^1^H-COSY spectrum, the correlation peaks between the above protons were also observed (Figure S4a). Based on the HSQC spectrum, their corresponding carbon signals were spotted at δc 131.2, 113.3, 117.9, 34.7, 15.6, and 24.2, respectively (Figure S4b). Previous studies have reported the presence of terpenoids, flavonoids, and phenolic acids in *O. dubium* [45,46,47]. Moreover, phenolic monoterpenes such as carvacrol and carvacrol acetate, as well as phenolic monoterpene glucosides (e.g., thymoquinol-2-*O*-β-glucopyranoside and thymoquinol-5-*O*-β-glucopyranoside), were found in this species [47].

**Figure 6:**
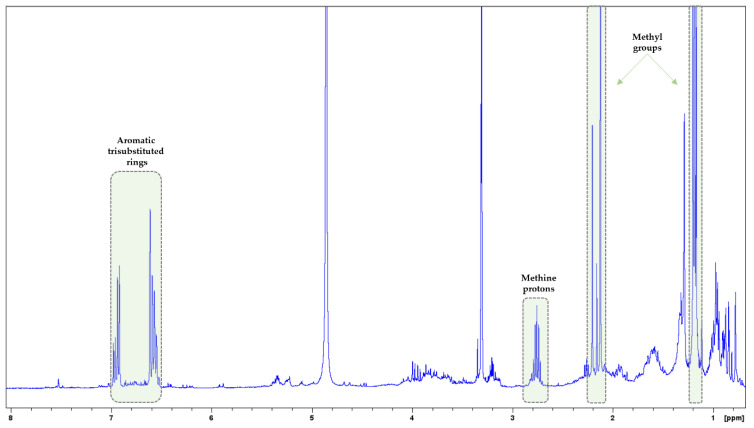
^1^H-NMR spectrum of *T. capitatus.* Signals of phenolic monoterpenes are indicated with green boxes.

In the NMR spectra of *T. capitatus*, signals from terpenoids, flavonoids, and phenolic acids were observed. Major signals of phenolic monoterpenes were also detected in its ^1^H-NMR spectrum: (i) aromatic protons of trisubstituted rings at δ_H_ range 7.00–6.50 ppm, (ii) methine protons at δ_H_ around 2.76, and (iii) methyl protons at δ_H_ 2.22–2.10 and 1.19–1.13 (Figure 6). In the ^1^H-^1^H-COSY spectrum, the correlation peaks between the above protons were also observed (Figure S5a). Their corresponding carbon signals were also observed in the HSQC spectrum (Figure S5b). In general, terpenoids, flavonoids, and phenolic acids have been reported in *T. capitatus* [48]. Phenolic monoterpenes, namely carvacrol and thymol, have also been found in *T. capitatus* extracts, apart from its essential oil [48].

**Figure 7:**
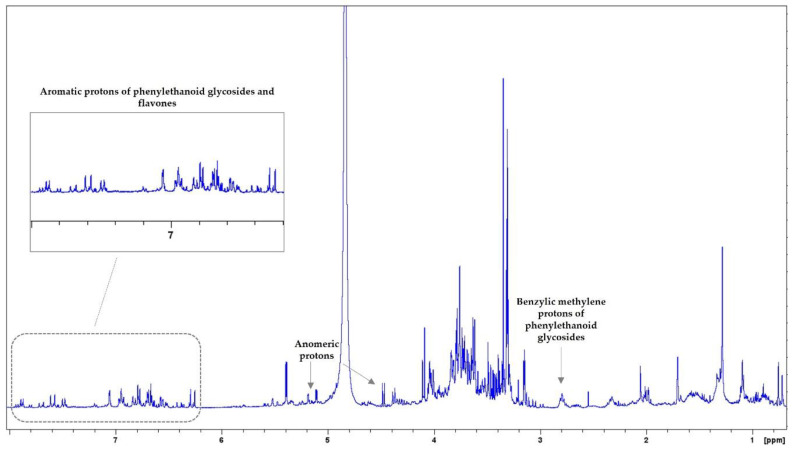
^1^H-NMR spectrum of *S.cypria.* Zoom-in of the aromatic region.

In the ^1^H-NMR spectrum of *S. cypria*, signals from terpenoids, flavonoids, and phenylethanoid glycosides were observed (Figure 7). In the upfield region (3.00–0.70 ppm), signals of terpenoids mainly appeared. The middle region (5.50–3.10 ppm) included signals, especially of sugars and their anomeric protons, as well as signals of iridoids. In the downfield region (7.90–6.20 ppm), signals of flavonoids (mainly flavones) and phenylethanoid glycosides were observed. At δ_H_ 7.60 (d, J = 16.2 Hz) and 6.28 (d, J = 16.2 Hz), *trans*-coupling olefinic signals ascribable to double-bond (HSQC: δc 147.5 and 114.4, respectively), at δ_H_ range 7.07–6.50 signals were observed that could belong to protons of aromatic trisubstituted rings, as well as at δ_H_ around 2.80–2.70 signals of benzylic methylene protons (HSQC: δc 36.3) were spotted. These assignments led to the assumption of the presence of phenylethanoid glycosides. In the ^1^H-^1^H-COSY spectrum, the principal correlation peaks among protons, which could belong to terpenoids, flavones, and phenylethanoid glycosides, were detected (Figure S6a). The HSQC spectrum is presented in Figure S6b. Genus *Sideritis* is rich in terpenes, flavonoids, phenylethanoid glycosides, and phenolic acids [49,50]. Among them, flavonoid glycosides (or/and acetylglycosides) and phenylethanoid glycosides are principal components of the genus *Sideritis*. Previous phytochemical studies in *S. cypria* extracts mentioned the presence of phytosterols (β-sitosterol and stigmasterol), diterpenes (ent-kaurane skeleton: sidol, isosidol, linearol, isolinearol, 3-acetyl-leucanthol), iridoids (e.g., 8-epiloganic acid, ajugoside, 7-*O*-acetyl-8-epi-loganic acid), flavone derivatives (mainly isoscutellarein acetylallosylglucosides and apigenin acylated derivatives), phenylethanoid glycosides (e.g., acteoside, leucosceptoside A, lavandulifolioside, leonoside A), and phenolic acids (such as chlorogenic acid and vanillic acid-4-*O*-β-D-glucopyranoside) [31,51,52].

### 3.3. Antioxidant Activity, Total Phenolics, and Flavonoid Content

The antioxidant activity of the extracts has been analyzed using a series of different assays. The highest activity (measured by DPPH and FRAP assays) was obtained for *M. piperita* extract, while *O. dubium* was found to be significantly stronger via the ABTS assay. The lowest antioxidant activity was noticed in the *S. cypria* extract. Even though our results show promising antioxidant activity of cultivated MAPs in Cyprus, the obtained values in this study are not comparable to those in the literature, as the tested extracts may differ in terms of the extraction conditions and the solvent used.

The results of the total polyphenol/flavonoid content along with the accompanying antioxidant activity are presented in Table 3. The ethanolic extracts from *M. piperita* exhibited the strongest antioxidant activity, followed by the highest amount of total phenolic and flavonoid compounds among the tested species. The total phenolic content of all the investigated extracts ranged from 91.32 to 196.96 µmol GAE/g DE. The maximum amount was recorded in *M. piperita* (followed by *O. dubium* and *T. capitatus*), while the lowest content was observed in *S. fruticosa*. A similar pattern was revealed for the flavonoids content as well, where the highest content of flavonoids (207.74 mg rutin/g DE) was obtained for *M. piperita* extract and the lowest (59.22 mg rutin/g DE) for *S. fruticosa* extract.

The antioxidant performance of the extract of *M. officinalis* appeared significantly higher than that of most of the tested species. It is reported that the plant is a rich source of antioxidants, particularly rich in phenolic compounds, comparable with known synthetic antioxidants [53]. Additionally, the flavonoid content of the tested Cypriot samples appeared higher (116.71 mg rutin/g DE) than other reports on ethanolic extracts (65.05 mg rutin/g DE) [54]. Spiridon et al. [55] reported even lower content in phenolics and flavonoids in *M. officinalis* dried extracts and moderate values compared to *Origanum vulgare* (higher content) and *Lavandula angustifolia* (lower content), which were studied together. Cypriot wild-growing oregano revealed a higher content of phenolic compounds (in the range of 230.96 to 730.66 µmol GAE/g DE) when other extracts were tested [47], compared to the ethanolic extracts of the cultivated plants in this study (162.02 µmol GAE/g DE). Flavonoid content, though, was higher in the cultivated plants (85.89 mg rutin/g DE) compared to the wild-growing samples, ranging from 18.61 to 70.15 mg rutin/g DE, depending on the solvent used. *S. cypria* is an endemic species from Cyprus, which is used as common mountain tea, as other *Sideritis* species in Balkan countries [51]. There are no reports that evaluate the antioxidant profile of the ethanolic extracts of *S. cypria* in the literature. As described by Lytra et al. [31], the antioxidant properties of the leaf and flower infusion and the decoction were lower than our findings in this study. The ethanolic extracts of *M. piperita* revealed the highest values among the tested plants in almost each assay. Previous reports on aquatic:methanolic extracts of vacuum-dried plants revealed a much lower content of total phenolics/flavonoids and antioxidant activity [56]. According to Dorman et al. [57], the choice of solvent used for the extraction may have a strong impact on the total phenolic content. Thus, *M. piperita* extracts obtained using a series of different solvents and their mixtures (not ethanol) turned out to vary within a high range of the total phenolics assay from 40.2 to 301.3 µmol GAE/g DE (for petroleum ether and ethyl acetate, respectively), while the highest value for flavonoids was extracted with methanol. The values that were obtained for the ethanolic extracts in the present work are within the reference range. As for the rest of the present results, the lowest values of total phenolic and flavonoid content, compared to the other tested species, were obtained for the sample of *S. fruticosa* (91.32 µmol GAE/g and 59.22 µmol rutin/g, respectively), which reflects its moderate antioxidant capacity as well. A study [58] that evaluated extracts from *S. fruticosa* using a series of solvents (but not ethanol) measured the total phenolic content of the aerial parts between 63.7 and 144.0 µmol GAE/g DE, while the value obtained for the ethanolic extract of this study was 91.31 µmol GAE/g DE. The extracts of *T. capitatus* exhibited moderate values in phenolic and flavonoid content compared to the other studied species, with 154.07 µmol GAE/g DE and 80.64 mg rutin/g DE, respectively. The acetonic and methanolic extracts of the species were examined by Benoutman et al. [59] and were found to be considerably lower than the ones from this study.

Therefore, the differences between the tested assays could be associated with the differences in the extraction protocols used in each study. Previous reports indicated that the different methods of extractions, namely infusion, maceration and ultrasound-assisted extraction and the obtained extracts may significantly affect the chemical profile and bioactivities of lemon balm (*Melissa officinalis* L.) extracts [60]. The importance of the antioxidant profile of MAPs relies particularly on the high chemical diversity of the wide spectrum of their biological activities, as will be described next, and that can be directly accredited to the origin and the cultivation/environmental conditions in which each MAP is growing.

### 3.4. Antimicrobial Activity

Results of antimicrobial activity indicate that all the tested extracts inhibit the growth of tested bacterial and fungal strains in different ranges of MIC and MBC/MFC values. The best antibacterial activity was achieved with *O. dubium* and *T. capitatus* extracts, but in some cases, the antibacterial potential of *S. cypria* was in the same value range as the tested oregano and thyme extracts (MIC 0.125 mg/mL, MBC 0.25 mg/mL). All the results obtained are comparable (in some cases even better) to the obtained values for positive controls E211 and E224, which may be of practical importance since these food preservatives are often associated with allergic reactions in sensitive individuals as well as many other side effects (Table 4) [61]. For example, in the case of *S. aureus* and *B. cereus*, the antibacterial potential of *O. dubium* was ~10 times higher for the MBC value (0.50 mg/mL) than that of E211 and E224 (4.00 mg/mL).

As for the antifungal activity, the results obtained are somewhat lower than the values obtained for the antibacterial assay, though they also highlight the potential of the tested extracts to be used as antifungal agents and are especially important in the control of fungi that grow on food/plant matrices after they have been stored for a while (Table 5). The most susceptible fungi to the activity of the tested samples were *P. funiculosum* and *P. verrucosum* var *cyclopium*, while the best activity was achieved with *O. dubium* and *S. cypria* extract (MIC 0.25 mg/mL, MFC 0.50 mg/mL). Moreover, the extract prepared from *M. officinalis* turned out to effectively retard the fungal growth of *A. versicolor* at MIC 0.25 mg/mL and MFC 0.50 mg/mL.

The results obtained in this study are quite in accordance with published data covering this topic in the last couple of years. Thus, according to Abdel-Naime et al. [62], the ethanolic extract of *M. officinalis* showed selective antimicrobial potential towards tested pathogenic microorganisms. The most susceptible bacteria to the activity of the extract turned out to be *Staphylococcus aureus* and *Pseudomonas aeruginosa* (MIC values in the range of 1.65 and 191.40 μg/mL), as well as the yeasts *Candida albicans*, *Candida krusei*, and *Candida glabrata* (MIC values in the range of 0.30–345.10 μg/mL). No activity was recorded towards *Escherichia coli* and *Klebsiella pneumoniae*, which highlights the results we obtained for *E. coli* (MIC 0.25 mg/mL, MBC 0.50 mg/mL). In a more recent review article covered by Petrisor et al. [63], the therapeutic properties of *M. officinalis* were validated, with available data on chemical constituents held responsible for its wide spectrum of biological applications.

As for the bioactive potential of *S. fruticosa*, Eltawaty et al. [64] demonstrated that the chloroform extract of the plant is highly efficient towards Gram-negative bacteria, including *E. coli* ATCC 25922, which is in accordance with our results obtained for the ethanolic extract. Polyphenolic compounds were identified as the main bioactive compounds in the extract, as is the case with our results. The in vitro antimicrobial potential of this plant was once again validated in a recent study by Dawra et al. [58]. Particularly, cold maceration in combination with cyclohexane, dichloromethane, ethyl acetate, and methanol resulted in high antioxidant (methanolic), antibacterial (methanolic and ethyl acetate), and acetylcholinesterase (ethyl acetate) activity of the tested extracts. All these data indicate that this plant has multiple medicinal and pharmaceutical potentials, which should be explored on a commercial scale.

Regarding the *T. capitatus* sample, our literature survey showed that despite its wide application in Moroccan cuisine and medicine on a daily basis, its chemical analysis and bioactive properties are yet insufficiently investigated. Thus, a comprehensive study by Benoutman et al. [59] showed that acetonic and methanolic extracts of *T. capitatus* have a high share of phenolic compounds. Furthermore, results obtained from the genotoxicity test revealed that the tested samples had no mutagenic potential but showed remarkable antifungal potential, especially at low doses, with *Microsporum canis* being the most susceptible to the activity of the extract. There is a link between phenolic compounds and the bioactivity of *T. capitatus*, which seems to be in accordance with our results since several studies previously showed that naturally occurring phenolic compounds exert strong activity towards a wide range of clinically significant pathogens linked to microbial infection [65].

Regarding *S. cypria*, taking into consideration that this taxon is narrowly endemic and its cultivation is not common, its wide application and overall use are limited. According to Lytra et al. [31], the methanol extract of the aerial parts showed high antioxidant and cytotoxic activities, whereas no antimicrobial activity was observed. In contrast, the present results of both antibacterial and antifungal activities were quite promising (Table 4). This was previously shown by Hanoğlu et al. [66] as well for the essential oil from *S. cypria*, which was active towards *C. albicans*, *E. coli*, and *S. aureus.* Findings obtained may be of great importance for the Cypriot market boost, as *S. cypria* can be further marketed, exported, and applied as a putative food preservative after approval in food sector research.

*M. piperita*, probably among the most commonly used species, has long been utilized for its bioactive properties. According to Indrayudha [67], the ethanolic extract of leaves has prominent antimicrobial potential, which, in combination with commercially available antibiotics, turned out to be exceptional (especially towards *K. pneumoniae*). Our results regarding the antibacterial potential of peppermint are consistent with these findings, since it was also demonstrated that it inhibits the growth of both Gram-positive and Gram-negative bacteria. The antimicrobial potential of *M. piperita* turned out to be highly applicable in veterinary practice as well. Along with rosemary and wind cheese, peppermint extracts inhibited the growth of 10 *E. coli* strains isolated from the feces of poultry. Keeping in mind that gastric upsets can be highly fatal in poultry farms the results obtained may be of high importance [68].

### 3.5. Reduced Cell Viability of Cancer Cell Lines following Treatment with the Plant Extracts

The ethanolic extracts of six plant species were tested for their effect on the cell viability of three female-derived cancer cell lines and two colon cancer cell lines. The specific cell lines were selected as representatives of the most commonly diagnosed types of cancer in women (breast cancer, ovarian cancer, and uterine cancer), while colon cancer cells were selected based on the fact that colon cancer ranks third among the cancer types with the highest mortality rates [69]. Thus, the female-derived MDA-MB231 (breast cancer cell line), HeLa (cervical cancer cell line), SkoV-3 (ovarian cancer cell line), and two colon cancer cell lines that differ in terms of their metastatic potential, namely the low-invasiveness HT-29 cells and the highly aggressive and metastatic HCT-116 colon cancer cells [70], were used. All cancer cells were subjected to a MTT cell viability assay following treatment with the ethanolic extracts for 48 and 72 h, as described in the Materials and Methods section. While the 48 h treatment did not have a significant effect on cell viability (see Table 6), the 72 h treatment showed a more prominent effect on all cell lines.

**Figure 8:**
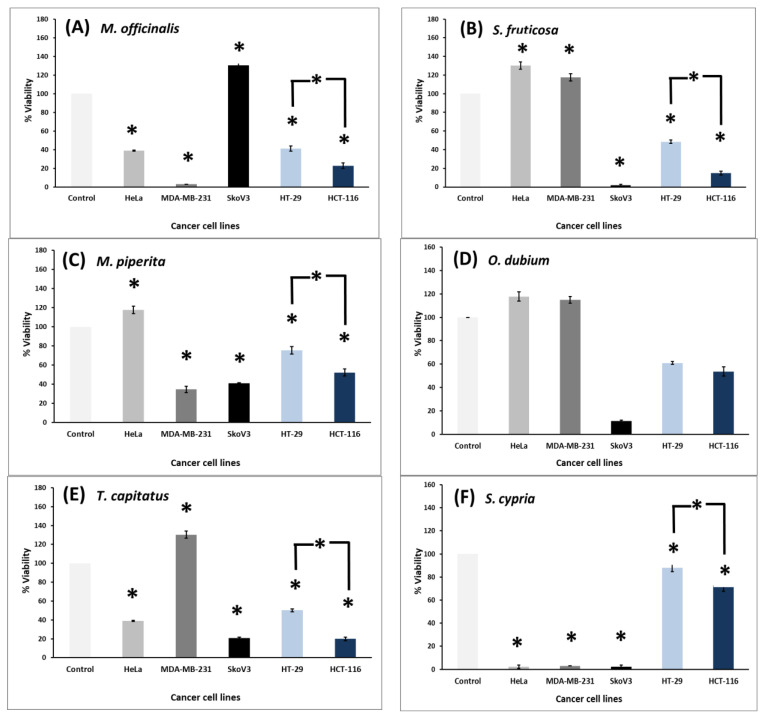
Graph representation of cell viability (%) in HeLa, MDA-MB231, SkoV3, HT-29, and HCT-116 cancer cell lines following treatment with ethanolic extracts from *M. officinalis* (**A**), *S. fruticosa* (**B**), *M. piperita* (**C**), *O. dubium* (**D**), *T. capitatus* (**E**), and *S. cypria* (**F**) at a concentration of 1000 μg/mL for 72 h. The MTT assay was performed in triplicate, and at least three independent experiments were performed for each extract. The graphs represent the mean value of the percent viability in the three independent experiments compared to the control (0.1% DMSO), which has 100% viability. All statistically significant changes (Student *t*-test, *p* value < 0.05) are indicated by an asterisk (*).

As shown in Figure 8, the viability of each one of the female-derived cancer cell lines was differently affected depending on the plant extract used for treatment. Specifically, the viability of HeLa cells was reduced after treatment with *M. officinalis*, while the effect was much more dramatic in MDA-MB231 breast cancer cells, whereas SkoV3 ovarian cancer cells seemed to be unaffected (Figure 8A). Treatment with *S. fruticosa* and *O. dubium* extracts (Figure 8B,D, respectively) seemed to have a more significant effect on reducing the viability of SkoV3 cells without affecting the viability of the other two female-derived cancer cell lines. Treatment with *M. piperita* extracts reduced cell viability in MDA-MB231 and SkoV3 cells without affecting HeLa cells (Figure 8C), while treatment with *T. capitatus* reduced viability of HeLa and SkoV3 cells without affecting MDA-MB231 cells. Interestingly, the plant extract of *S. cypria* was the only one that induced a dramatic reduction in cell viability against all tested female-derived cancer cells (Figure 8F). A previous study that evaluated the effect of 44 plants from traditional Thai remedies on the cell viability of cells from the three main cancer types occurring in women (breast, ovarian, and cervical) showed that 17 out of 44 were food ingredients in Thai cuisine, nine of which significantly reduced cell viability [71]. Similar to our results, *S. cypria* extract can also be used as a food ingredient in our traditional cuisine.

As for the effect of treatment with the plant extracts on the two colon cancer cell lines, as shown in Figure 8, treatment of cells with all six extracts significantly reduced cell viability in both cell lines (Figure 8A–F, compare the last two blue bars to the first light gray bar), indicating that all six extracts demonstrated cytotoxic activity. Most importantly, though, extracts from *M. officinalis* (Figure 8A), *S. fruticosa* (Figure 8B), *M. piperita* (Figure 8C), *T. capitatus* (Figure 8E), and *S. cypria* (Figure 8F) exhibited a stronger effect in the highly invasive HCT-116 cell line (compare dark blue bar with light blue bar). However, this was not the case for *O. dubium* (Figure 8D), where the extract had an equivalent effect on both cell lines.

The IC_50_, which denotes the concentration at which 50% of cell proliferation and growth is inhibited, was also calculated for all extracts, and the results are shown in Table 6. Overall, the plant extracts exhibited reduced IC_50_ values on human cancer cell lines 72 h after treatment compared to 48 h after treatment, indicating higher anti-proliferative activity. Cell viability of the extracts from *S. cypria* in all the female-derived cancer cell lines, as well as the extracts from *M. officinalis*, *S. fruticosa*, *M. piperita*, and *T. capitatus* in HT-29 and HCT-116 colon cancer cells, is verified by the IC_50_ values. For instance, the maximum anticancer activity of the plant extract of *S. cypria* was recorded with MDA-MB 231 (IC_50_ = 166.8 µg/mL), followed by HeLa (IC_50_ = 264.3 µg/mL) and SkoV3 (IC_50_ = 352.3 µg/mL). Moreover, the strong effect of *M. officinalis* extract on cell viability of MDA-MB 231 cells is verified with the reduced IC_50_ value (IC_50_ = 181.3 µg/mL) (Table 6).

Regarding the colon cancer cell lines, the IC_50_ values for *O. dubium* and *S. cypria* could not be calculated. However, the IC_50_ value for *T. capitatus* extract was four times less in highly metastatic HCT-116 cells compared to the less metastatic HT-29 cells. The IC_50_ value for *M. officinalis* extract in HCT-116 cells was half of the respective value in HT-29 cells, while the IC_50_ value for *M. piperita* extract could only be calculated in HCT-116 cells. Finally, the IC_50_ value for *S. fruticosa* extract in HCT-116 cells was eight times less than the respective value in HT-29 cells. This is of utmost biological and clinical significance as it indicates that more aggressive and metastatic cells (i.e., HCT-116 cells) are more sensitive to the plant extracts from *T. capitatus*, *M. officinalis*, *M. piperita*, and *S. fruticosa* and are therefore easier to eliminate than the less metastatic cancer cells (HT-29 cells). Consequently, these plant extracts have the potential to be further exploited therapeutically to target metastatic tumors.

Overall, the present findings indicate that treatment of cells with all six extracts significantly reduced cell viability in both colon cancer cell lines (Figure 8A–F), highlighting that all six extracts have cytotoxic activity. Contrary to this, each treatment affected the viability of female-derived cancer cells differently. The most common cancers occurring in women are breast, cervical, and ovarian cancer, with the first being the leading cause of death among women worldwide [72]. Comparing the present results for these three female-derived cancer cells, it may be concluded that the viability of SkoV3 cells was the only one affected by all the plant extracts tested, except for *M. officinalis*. This result indicates that ovarian cancer cells might be more sensitive to plant extract treatments compared to breast and cervix cancer cells. However, *M. officinalis* extracts dramatically reduced the viability of MDA-MB231 cells. The effect of *M. officinalis* on the cell viability of MCF-7 breast cancer cells was shown to be through apoptosis and the autophagy cell death pathway [73]. Therefore, it would be interesting for future studies to elucidate the antitumor mechanism of action in MDA-MB231 cells. Interestingly, the effect of the ethanolic extract from *S. cypria* reduced cell viability in all the female-derived cancer cells. According to Lytra et al. [31], the methanolic extract of *S. cypria* did not show any effect on the cell viability of MDA-MB231 cells; however, groups of *S. cypria* with phytosterols, diterpenoids, and apigenin derivatives exerted cytotoxic effects, and they induced a significant decrease in the cell viability of the MDA-MB231 cell line in a concentration-dependent manner [74].

More importantly, the effects of extracts from *T. capitatus*, *M. officinalis*, *M. piperita*, and *S. fruticosa* were more prominent in reducing the cell viability of the highly metastatic HCT-116 cell line, and these results were further supported by the actual IC_50_ values (Table 6), where the IC_50_ value was up to eight times higher in the less metastatic cells compared to the more metastatic ones. This indicates that these extracts act more potently and in lower concentrations in highly metastatic cells, which means that they have enormous anti-cancer potential as they can specifically target and eliminate highly aggressive and metastatic cells while sparing less aggressive neighboring cells. This basically addresses the most challenging problem in developing new anti-cancer therapies: how to specifically target and kill cancer cells without destroying normal adjacent tissues or other normal cells in the body. Hence, having a naturally occurring plant extract target highly metastatic cells at a low concentration that does not affect non-metastatic cells or normal cells will essentially be revolutionary for cancer therapy research, and given the accessibility of the extracts, this may be easily transferable to the clinic. However, this was less accurate for extracts from *S. cypria*, which, although showing the same tendency, did not have a difference in IC_50_ value that could be calculated. Finally, the extract of *O. dubium* was the only one that did not show any difference between the two colon cancer cell lines. This finding further supports the specificity and importance of the effects of the other four extracts, which hold immense therapeutic potential and are definitely worth investigating in future studies. It would also be interesting to compare the effect of these extracts on their respective normal cells as well.

Overall, the sustainability and security of the food supply on a global scale are fundamentally dependent on crop protection and preservation. Food waste brought on by microbial development, environmental factors, and insect infestation has been successfully prevented by several preservation techniques. Yet, research has shown that using synthetic biocides and preservatives to protect and preserve crops poses serious health risks. Therefore, the findings of the present study may have multiple applications, including in the food industry, among others, based on the antimicrobial and antioxidant activity of the MAP extracts. The main reason for this is that oxidative damage and microbial spoilage are the main causes of food deterioration, followed by great financial losses.

## 4. Conclusions

In the present study, six medicinal/aromatic plants (*S. cypria*, *O. dubium*, *M. officinalis*, *M. piperita*, *T. capitatus*, and *S. fruticosa*) cultivated in Cyprus were investigated for their phytochemical composition, nutritive and biological activities, including antioxidant and antimicrobial effects, as well as their effect on cell viability. Plants were rich in minerals, with *M. piperita* demonstrating high levels of N and Mg, while *S. cypria* had high levels of K, Na, P, and Ca. All six species exhibited a high content of total phenols, flavonoids, and antioxidant capacity, with *M. piperita*, followed by *O. dubium* and *T. capitatus* having the highest levels. The main chemical categories in each studied plant extract were identified by NMR analysis, revealing (i) triterpenoids and hydroxycinnamic acid derivatives in *M. officinalis*; (ii) terpenoids, flavonoids, and phenolic acid derivatives in *S. fruticosa*; (iii) flavonoids (especially flavanone) and phenolic acid derivatives in *M. piperita*; (iv) phenolic monoterpenes in *O. dubium* and *T. capitatus*; and (v) terpenoids, flavones, and phenylethanoid glycosides in *S. cypria*. High antibacterial activity was found in *O. dubium* and *T. capitatus*, while *O. dubium* and *S. cypria* revealed great antifungal properties. The examined species induced a significant reduction in cell viability in female-derived cancer cells, with an interesting observation for the *S. cypria* extracts. Interestingly, extracts from *T. capitatus*, *M. officinalis*, *M. piperita*, and *S. fruticosa* had a greater effect on the cell viability of the highly metastatic HCT-116 cell line, indicating immense potential for exploiting them therapeutically to treat higher-grade, more aggressive, and metastatic colorectal cancer tumors. The results obtained in this research indicate the high efficiency of MAP extracts in several aspects of interest. They suggest that the use of natural products as safe and affordable ingredients without negative effects on consumer health or the environment is quite possible, and that the food and pharmaceutical industries should invest on a larger scale in the development of MAP-based pharmaceuticals and food preservatives.

## Figures and Tables

**Table 1:** Mineral analysis of the aerial parts of the tested medicinal and aromatic plant (MAP) species. Data are presented as the mean of four replicates ± SE. Values in the columns for each tested species followed by the same letter are not significantly different; *p* ≤ 0.05.

MAP Species	Nitrogen (N) g/kg	Sodium (Na) g/kg	Potassium (K) g/kg	Magnesium (Mg) g/kg	Calcium (Ca) g/kg	Phosphorus (P) g/kg
*Melissa officinalis*	15.42 ± 0.55 bc	0.13 ± 0.04 d	22.17 ± 0.15 a	4.22 ± 0.06 b	9.20 ± 0.13 d	2.58 ± 0.04 bc
*Salvia fruticosa*	16.46 ± 0.76 b	0.59 ± 0.04 c	15.20 ± 0.44 c	3.12 ± 0.12 c	9.54 ± 0.28 d	1.57 ± 0.11 d
*Mentha piperita*	20.55 ± 0.31 a	0.66 ± 0.02 c	16.77 ± 0.15 b	5.82 ± 0.04 a	14.20 ± 0.22 b	2.43 ± 0.28 c
*Origanum dubium*	11.46 ± 0.28 d	0.13 ± 0.01 d	12.69 ± 0.21 d	4.31 ± 0.21 b	10.22 ± 0.39 d	2.99 ± 0.09 b
*Thymus capitatus*	14.19 ± 0.13 c	1.01 ± 0.03 b	15.19 ± 0.28 c	2.09 ± 0.05 d	11.46 ± 0.22 c	1.37 ± 0.05 d
*Sideritis cypria*	13.67 ± 0.10 c	1.28 ± 0.02 a	22.07 ± 0.15 a	2.30 ± 0.08 d	17.49 ± 0.18 a	3.75 ± 0.05 a

**Table 2:** Nutritional value of the aerial parts of the tested medicinal and aromatic plant (MAP) species. Data are presented as the mean of four replicates ± SE. Values in the columns for each tested species followed by the same letter are not significantly different; *p* ≤ 0.05.

MAP Species	Dry Matter %	Humidity %	Ash %	Total Fats %	Proteins %	Carbohydrates %	Energy kcal/100 g
*Melissa officinalis*	21.63 ± 0.36 c	78.36 ± 0.36 a	8.18 ± 0.36 b	1.71 ± 0.04 d	9.64 ± 0.69 bc	80.45 ± 0.48 b	375.86 ± 1.39 c
*Salvia fruticosa*	29.36 ± 0.43 c	70.63 ± 0.43 b	6.97 ± 0.19 b	5.89 ± 0.02 a	10.28 ± 0.96 b	76.84 ± 0.38 c	401.54 ± 0.65 a
*Mentha piperita*	19.14 ± 0.57 c	80.85 ± 0.57 a	11.28 ± 0.21 a	2.81 ± 0.01 c	12.84 ± 0.39 a	73.06 ± 0.26 d	368.94 ± 0.82 d
*Origanum dubium*	21.12 ± 1.22 c	78.87 ± 1.22 a	7.02 ± 0.66 b	4.03 ± 0.06 b	7.16 ± 0.35 d	81.78 ± 0.63 b	392.07 ± 2.61 b
*Thymus capitatus*	42.64 ± 0.79 a	57.35 ± 0.79 c	6.30 ± 0.56 b	0.69 ± 0.01 e	8.87 ± 0.16 c	84.12 ± 0.61 a	378.23 ± 2.27 c
*Sideritis cypria*	28.90 ± 0.16 b	70.76 ± 0.30 b	11.84 ± 0.95 a	2.92 ± 0.29 c	8.54 ± 0.11 c	76.68 ± 0.44 c	367.26 ± 1.12 d

**Table 3:** Total phenolic (as gallic acid equivalent; GA) and flavonoid (as rutin equivalent) content and antioxidant (DPPH, FRAP, ABTS; as trolox equivalent) activity of the ethanolic extracts of the tested medicinal and aromatic plant (MAP) species. Data are presented as the mean of four replicates ± SE on a dry extract (DE) weight basis. Values in the columns for each tested species followed by the same letter are not significantly different; *p* ≤ 0.05.

MAP Species	Total Phenolic Content (µmol GA/g DE)	DPPH (mg Trolox/g DE)	FRAP (mg Trolox/g DE)	ABTS (mg Trolox/g DE)	Total Flavonoid Content (mg Rutin/g DE)
*Melissa officinalis*	138.37 ± 0.65 d	195.02 ± 3.22 b	262.65 ± 1.78 b	188.87 ± 2.98 d	116.71 ± 1.58 b
*Salvia fruticosa*	91.32 ± 1.09 f	91.90 ± 1.46 c	182.03 ± 1.29 d	171.48 ± 3.72 e	59.22 ± 0.25 e
*Mentha piperita*	196.96 ± 1.73 a	295.18 ± 3.79 a	389.63 ± 1.27 a	252.41 ± 1.34 c	207.74 ± 1.93 a
*Origanum dubium*	162.02 ± 2.32 b	90.12 ± 1.04 c	206.84 ± 2.60 c	427.86 ± 1.13 a	85.89 ± 1.88 c
*Thymus capitatus*	154.07 ± 0.77 c	71.66 ± 1.43 d	210.84 ± 1.87 c	351.75 ± 4.64 b	80.64 ± 0.97 c
*Sideritis cypria*	100.74 ± 0.93 e	79.25 ± 2.29 d	160.02 ± 2.49 e	155.99 ± 4.39 e	72.52 ± 0.55 d

**Table 4:** Antibacterial (minimal inhibitory concentration-MIC; minimal bactericidal concentration-MBC) activity of the tested extracts (mg/mL).

		*S. aureus* (ATCC 11632)	*B. cereus* (Clinical Isolate)	*L. monocytogenes* (NCTC 7973)	*S. enterica* subsp. *enterica* serovar Typhimurium (ATCC 13311)	*E. coli* (ATCC 25922)
*M. officinalis*	MIC	0.25	0.25	0.25	0.50	0.25
	MBC	0.50	0.50	0.50	1.00	0.50
*S. fruticosa*	MIC	0.50	0.25	0.25	0.25	0.25
	MBC	1.00	0.50	0.50	0.50	0.50
*M. piperita*	MIC	0.50	0.50	0.50	0.50	0.25
	MBC	1.00	1.00	1.00	1.00	0.50
*O. dubium*	MIC	0.25	0.25	0.125	0.125	0.125
	MBC	0.50	0.50	0.25	0.25	0.25
*T. capitatus*	MIC	0.50	0.25	0.125	0.125	0.125
	MBC	1.00	0.50	0.25	0.25	0.25
*S. cypria*	MIC	0.50	0.25	0.125	0.125	0.125
	MBC	1.00	0.50	0.25	0.25	0.25
E211	MIC	4.00	0.50	1.00	1.00	1.00
	MBC	4.00	0.50	2.00	2.00	2.00
E224	MIC	1.0	2.0	0.5	1.0	0.5
	MBC	1.0	4.0	1.0	1.0	1.0

**Table 5:** Antifungal (minimal inhibitory concentration (MIC); minimal fungicidal concentration (MFC)) activity of the tested extracts (mg/mL).

		*A. fumigatus* (ATCC 9197)	*A. versicolor* (ATCC 11730)	*A. niger* (ATCC 6275)	*P. funiculosum* (ATCC 36839)	*P. verrucosum* var. *cyclopium* (Food Isolate)
*M. officinalis*	MIC	0.50	0.25	0.50	1.00	1.00
	MFC	1.00	0.50	1.00	2.00	2.00
*S. fruticosa*	MIC	1.00	1.00	0.50	1.00	1.00
	MFC	2.00	2.00	1.00	2.00	2.00
*M. piperita*	MIC	0.50	0.50	0.50	1.00	1.00
	MFC	1.00	1.00	1.00	2.00	2.00
*O. dubium*	MIC	0.50	1.00	0.50	0.25	0.50
	MFC	1.00	2.00	1.00	0.50	1.00
*T. capitatus*	MIC	0.50	1.00	1.00	0.50	0.50
	MFC	1.00	2.00	2.00	1.00	1.00
*S. cypria*	MIC	0.50	0.50	0.25	0.25	0.25
	MFC	1.00	1.00	0.50	0.50	0.50
E211	MIC	1.00	2.00	1.00	1.00	2.00
	MBC	2.00	4.00	2.00	2.00	4.00
E224	MIC	1.00	1.00	1.00	0.50	1.00
	MBC	1.00	1.00	1.00	0.50	1.00

**Table 6:** Half maximal inhibitory concentration (IC_50_) calculated for all cell lines treated with the six extracts for 72 h with a concentration of 1000 μg/mL.

	Female-Derived Cancer Cells			Colon Cancer Cells	
Treatment: 1000 μg/mL of plant extracts for 72 h	**MDA-MB231**	**SkoV3**	**HeLa**	**HT-29**	**HCT-116**
*Origanum dubium*		448.6		>1000	>1000
*Sideritis cypria*	166.8	352.3	264.3		
*Thymus capitatus*		394.2	698.4	858.2	237.5
*Melissa officinalis*	181.3		676.2	765.5	371.2
*Mentha piperita*	642.3	756.5			225.6
*Salvia fruticosa*		184.9		814.4	106.3

## Data Availability

Our data are not directly available, as we have calculations, and data that we need to protect from the general readers. If a reader want to get in contact with us, please contact the corresponding authors.

## Supplementary Materials

The following supporting information can be downloaded at: https://www.mdpi.com/article/10.3390/biology13010045/s1, Figure S1–S6: Two-dimensional NMR spectra (COSY and HSQC).
