# Supplementary material for: Phytochemical Profiles and Biological Activities of Plant Extracts from Aromatic Plants Cultivated in Cyprus
Source: Biology (Basel). 2024 Jan 15;13(1):45. doi: 10.3390/biology13010045 (PMC10813336; doi:10.3390/biology13010045)
Supplement: Supplementary file 1 [file biology-13-00045-s001.zip › biology-2757432-supplementary.pdf]

Supplementary material

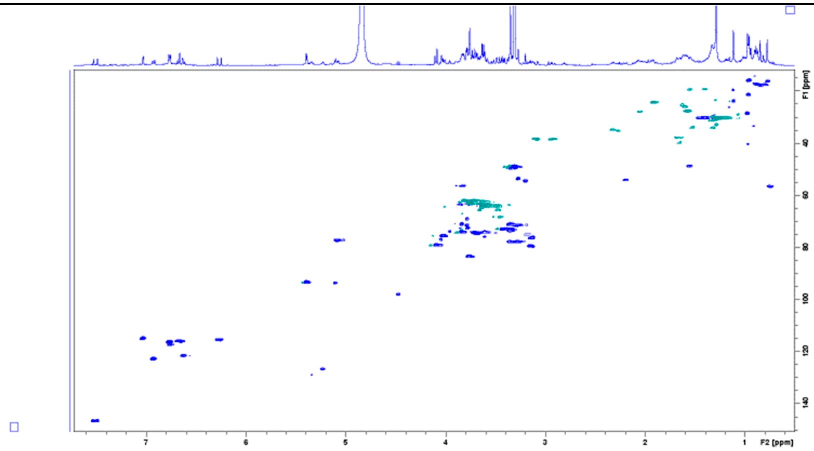

Figure S1a. HSQC spectrum of *M. officinalis*

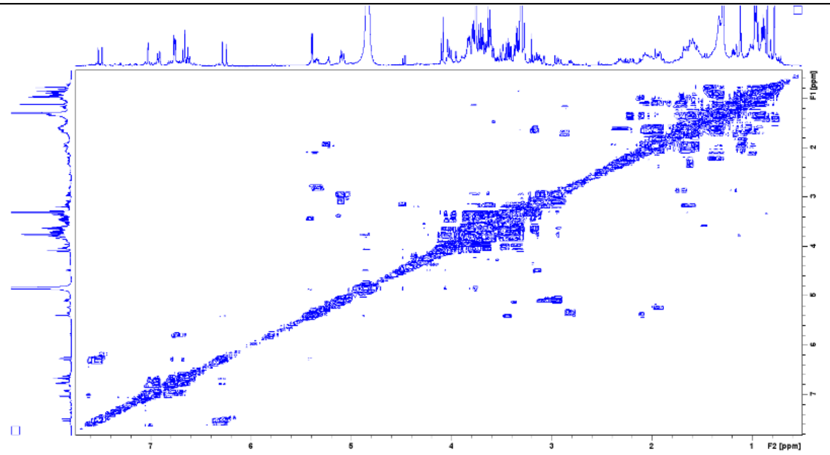

Figure S1b. ^{1}H-^{1}H-COSY spectrum of *M. officinalis*

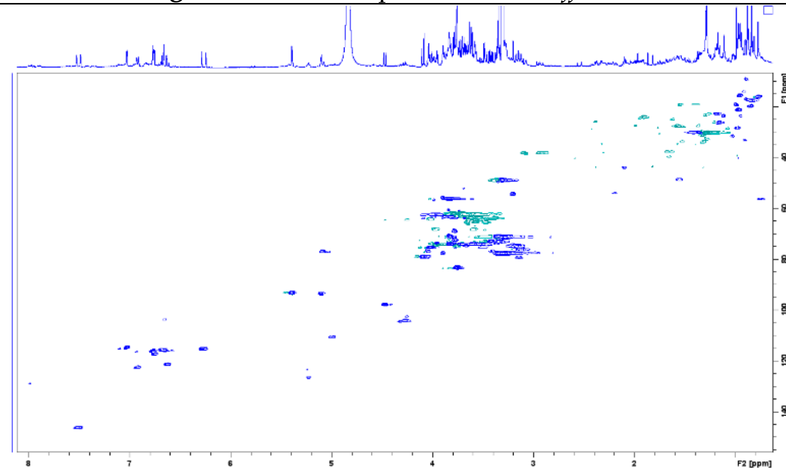

Figure S2a. HSQC spectrum of *S. fruticosa*

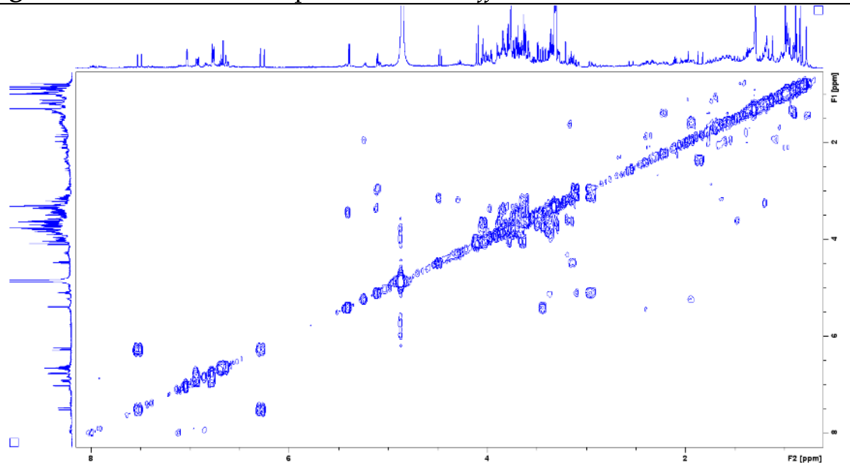

Figure S2b. ^{1}H-^{1}H-COSY spectrum of *S. fruticosa*

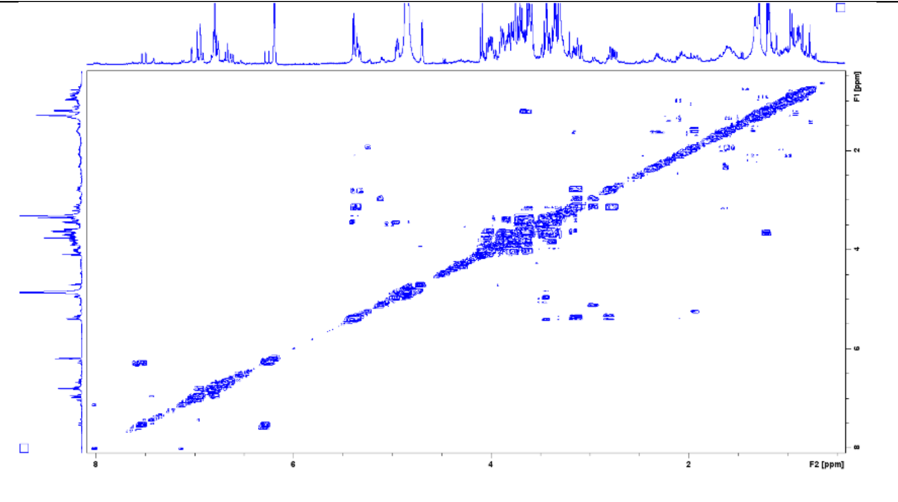

Figure S3a. ¹H-¹H-COSY spectrum of *M. piperita*

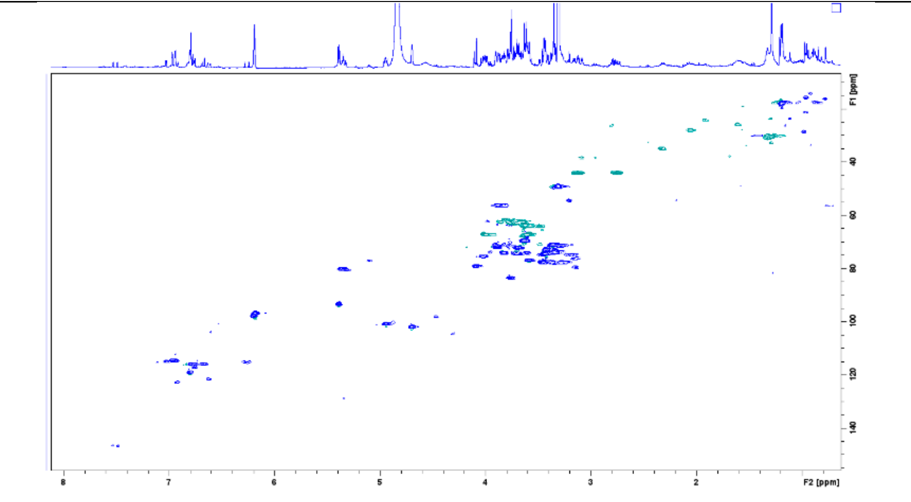

Figure S3b. HSQC spectrum of *M. piperita*

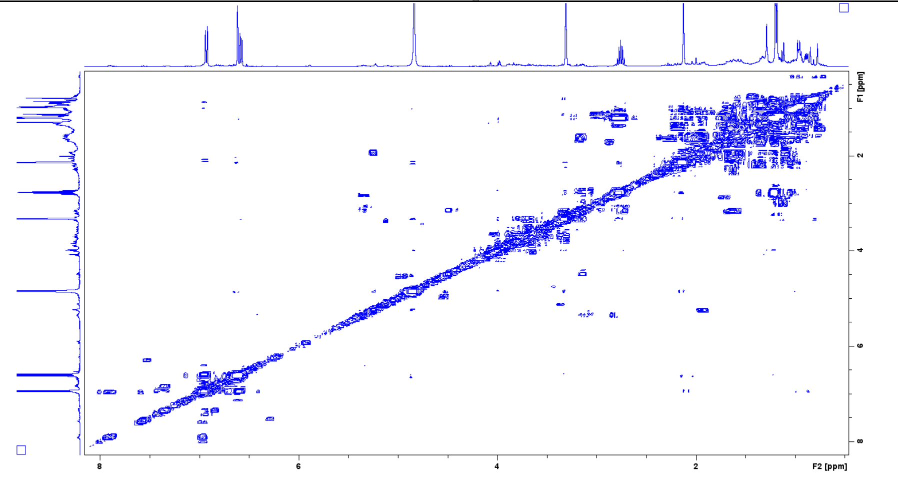

Figure S4a. ¹H-¹H-COSY spectrum of *O. dubium*

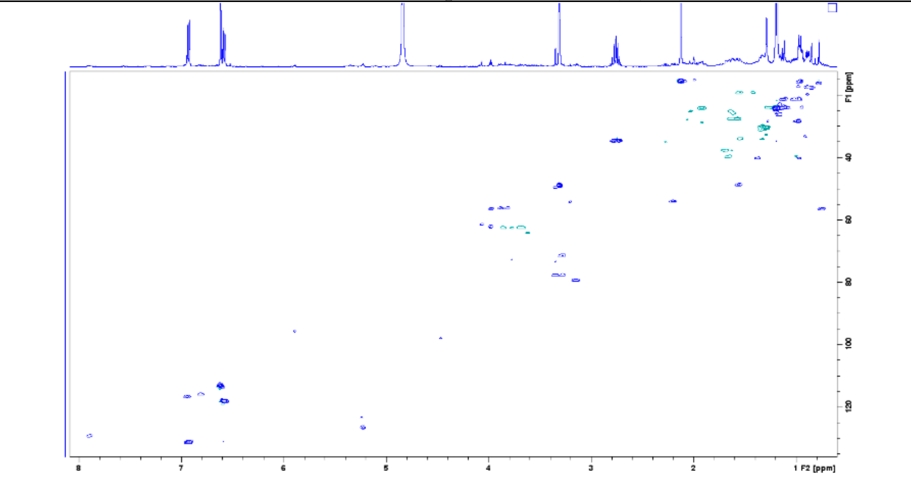

Figure S4b. HSQC spectrum of *O.dubium*

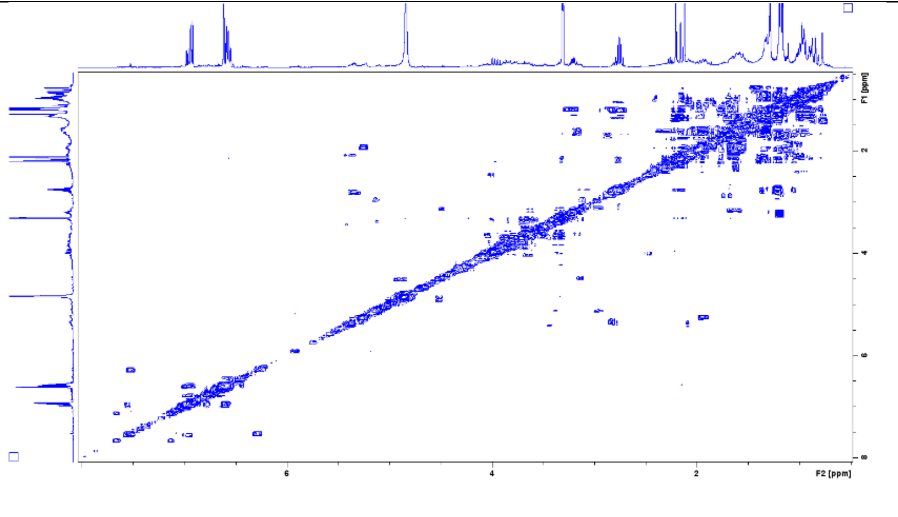

Figure S5a. ^{1}H-^{1}H-COSY spectrum of *T. capitatus*

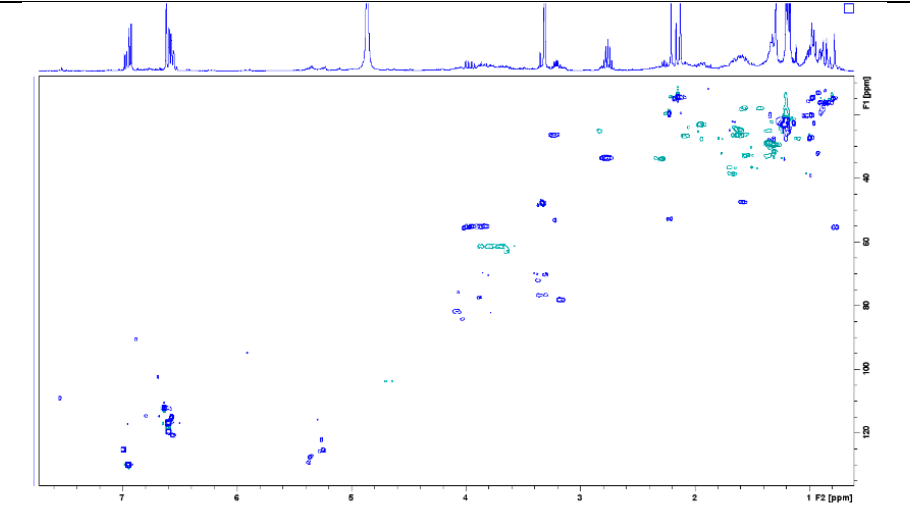

Figure S5b. HSQC spectrum of *T. capitatus*

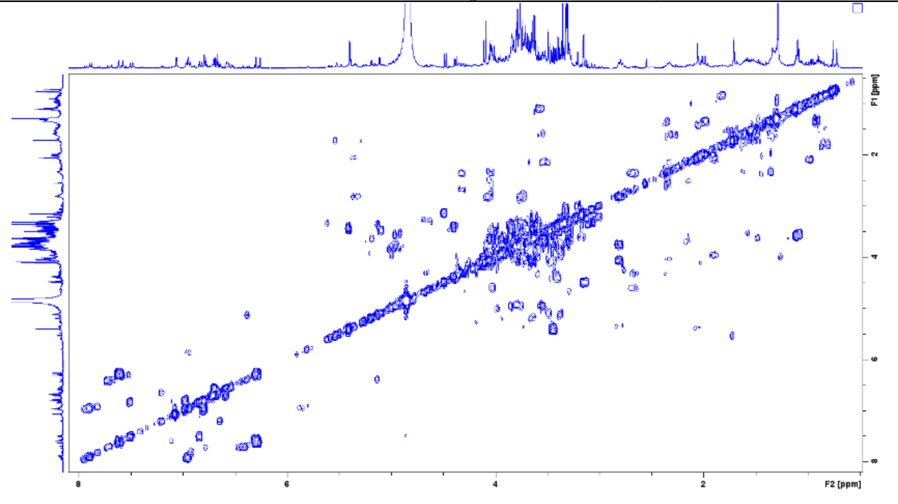

Figure S6a. ^{1}H-^{1}H-COSY spectrum of *S. cypria*

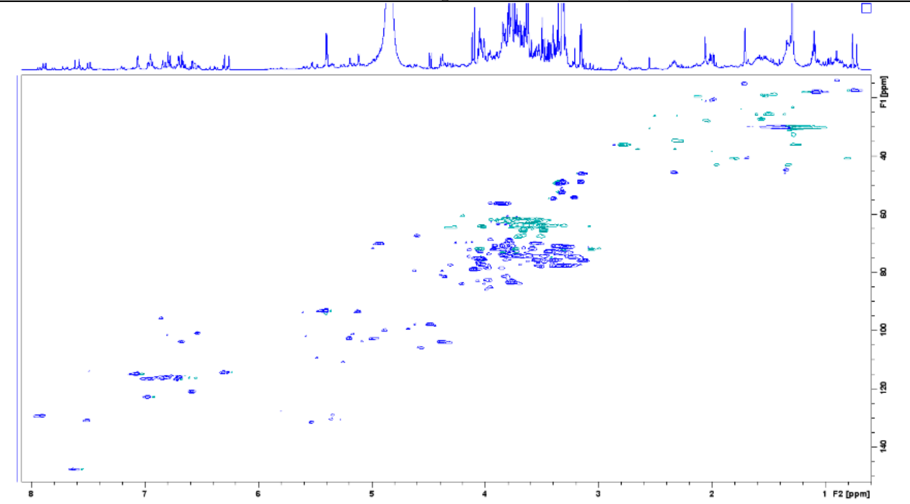

Figure S6b. HSQC spectrum of *S. cypria*
